# Interactive effects of dietary protein and nano-chitosan on growth performance, immune response, and histological aspects of lymphoid organs in broiler chickens

**DOI:** 10.1007/s11250-023-03855-2

**Published:** 2024-01-27

**Authors:** A.H. Mansour, M.H. Rabie, Eman A. El-Said, Hayam M. Abo El-Maaty

**Affiliations:** 1 Poultry Production Department, Faculty of Agriculture, Mansoura University, Mansoura, Egypt; 2 Poultry Production Department, Faculty of Agriculture, Damietta University, Damietta, Egypt

**Keywords:** Plant protein sources, Nano-chitosan, Performance, Immunity, Blood parameters, Broiler chickens

## Abstract

A feeding trial was conducted to assess the effect of partial replacement of dietary soybean meal by three plant protein sources: coconut, rocket seed, and black cumin meals with their combination in the presence or absence of nano-chitosan (NCH) on growth performance and immune response in broiler chickens. Five starter and grower diets were formulated and used from 1 to 42 days of age. The NCH was added to starter and grower diets at 1.0 g/kg. Five-hundred-fifty-day-old Arbor Acres Plus broiler chicks were randomly divided into ten treatments with five equal replications. Final body weight (FBW), body weight gain (BWG), feed intake (FI), feed conversion ratio (FCR), and blood plasma parameters were investigated. Histological aspects of lymphoid organs (thymus: T, bursa of Fabricius: B, and spleen: S) were characterized. Apart from added NCH, the FBW, BWG, and FCR of broilers fed the diets containing the tested plant proteins were significantly superior to the control ones. However, FI of birds fed the diets containing CM alone or combined with RSM plus BCM was significantly reduced. All experimental broilers displayed high plasma levels of IgG compared with the control group. There were significant increases in plasma concentrations of IgM, IgA, and T4 for groups that fed the diets containing RSM, BCM, and mixture of CM, RSM, and BCM compared with their controls. The T3 levels of broilers fed the tested plant proteins were significantly increased compared with the controls. Aside from plant protein source, broilers fed the NCH-enriched diets achieved significant increases in levels of IgM, TAC, and FSH and activities of CAT and SOD but reduced the MDA level compared with control. The interactions between plant protein source and added nano-chitosan were not interrelated. Furthermore, CM, RSM, and BCM can be used as complementary dietary proteins singly or combined with NCH with no adverse effects on growth performance. Addition of NCH molecules has a positive effect on live body weight and increases feed intake compared with control chicks.

**Graphical Abstract:**

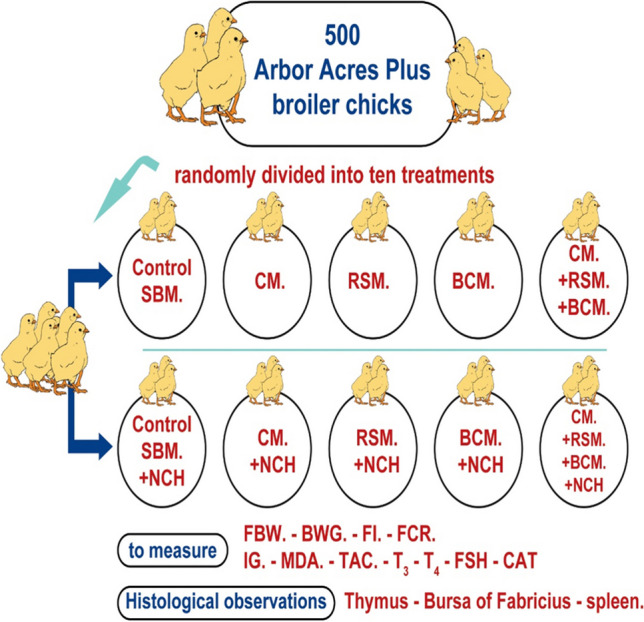

**Supplementary Information:**

The online version contains supplementary material available at 10.1007/s11250-023-03855-2.

## Introduction

Protein is the most important component in poultry diets, particularly in countries where protein-rich feed ingredients are limited (Beski et al. 2015). When formulating broiler rations, the main emphasis is focused on their crude protein content, because protein is the critical component of poultry diets, and together with other essential nutrients (such as carbohydrates, lipids, vitamins, minerals, and water) is vital for life (Cheeke 2005). Formulating broiler diets based on corn and soybean meal (SBM) has increased the demand for soybean meal, and this leads to increasing the feeding cost and total cost of poultry production. There are two possible approaches for lowering broiler chick feed costs. The first approach is alternative usage of inexpensive plant protein sources such as coconut meal (also called copra meal), rocket seed meal, and black cumin meal. The second approach is to improve health, immune status, and growth performance of broilers by means of the natural antioxidants and natural growth promoters.

Coconut meal (CM) is made up of coconut residues after oil extraction, drying, and grinding. Previous studies have demonstrated that its crude protein (CP) and ether extract (EE) concentrations (on dry matter basis) vary between 20.9 and 24.3% and 1.9 and 8.4%, respectively (NRC 1994; Sundu et al. 2009; Son et al. 2012; Son et al. 2017). The crude fiber (CF) content of CM was reported to range from 7 to 15% (Sundu et al. 2009). Therefore, the use of CM as an effective alternative protein source for poultry is limited due to its poor palatability (Sundu et al. 2009), high CF content (Kim et al. 2001; Sundu and Dingle 2003; Siebra et al. 2008), poor amino acid profile (Saittagaroon et al. 1983; Sundu et al. 2009), and certain physicochemical properties (Sundu et al. 2009; Diarra et al. 2018). The production of rocket seed meal (RSM) and black cumin meal (BCM) in Egypt has been steadily increased because of the strong demand to the volatile oils for pharmaceutical purpose. In this regard, RSM was found to be an effective replacement for up to 32% of SBM in growing Japanese quail diets (El-Shafei et al. 2007), with no negative effects on growth performance, carcass characteristics, or blood constituents. They also claimed that RSM, due to its antioxidant properties, can act as an immunostimulant. Abo El-Maaty (2009) reported that hens fed the RSM-containing diets displayed no significant differences in FI, BWG, or FCR compared with the control group. These plant protein sources, on the other hand, act as immunostimulants due to their antioxidant properties (Abo El-Maaty, 2009). Chitosan nanoparticles outperform ordinary chitosan in terms of activity because nanonization has many advantages such as increasing compound solubility and improving absorbability (Wen et al. 2011). Quite predictably, nano-chitosan (NCH) has been shown to have immune-boosting, antimicrobial, and anticancer properties (Iqbal et al. 2003). It may also act as an adjuvant by promoting endocytic uptake and increasing immune responses. Furthermore, NCH has lower cytotoxicity and greater stability (Neimert-Andersson et al. 2011). The goal of this study was to see how three plant protein sources and their combination in the presence or absence of nano-chitosan affected growth performance, immune response, selected blood biochemical parameters, and histological characterization of lymphoid organs in broiler chickens.

## Materials and methods

### Experimental design, birds and diets

The current study was carried out at a private poultry farm in Dekernis City, Dakahlia Governorate, El-Mansoura, Egypt, during the period from February to March 2019. Five-hundred-fifty-day-old Arbor Acres Plus broiler chicks were used. At the first day of age, birds were randomly divided into ten equal experimental groups. Each experimental group was subdivided into five replications of eleven chicks each. Each replication of chicks was kept in a compartment of floor pens with dimensions of 125 × 125 cm with stocking density of 11 chicks, supplied with an artificial source of light to provide a daily photoperiod of about 16 h. A completely randomized design in a factorial arrangement of treatments (5 × 2) was used in this study; five dietary protein sources [soybean meal (SBM), coconut meal (CM), rocket seed meal (RSM), black cumin meal (BCM), and a mixture of CM, RSM, and BCM] with or without nano-chitosan supplementation. Five isocaloric and isonitrogenous starter (about 13.18 MJ ME/kg and 23% CP ) and grower (about 13.18 MJ ME/kg and 20% CP) diets were created and implemented. The NCH was added to starter and grower diets at 1.0 g/kg. All chicks had free access to a mash feed and freshwater throughout the experimental period, from 1 to 6 weeks of age. Composition of starter and grower diets and calculated analysis are given in Table 1. These diets were formulated based on the nutrient contents of CM, RSM, and BCM, determined by the official methods of analysis (AOAC 1990) and the nutritional requirements of broiler chickens, recommended by the National Research Council (NRC 1994).

**Table 1:** Composition and calculated analysis of starter and grower diets of different plant protein sources fed to broiler chickens during the fattening period

Ingredients (%)	Starter diets (0–3 weeks)					Grower diets (3–6 weeks)				
	Control	CM	RSM	BCM	CM + RSM + BCM	Control	CM	RSM	BCM	CM + RSM + BCM
Yellow corn, ground	61.50	58.40	59.40	59.50	58.99	67.20	64.20	64.70	65.20	64.89
Soybean meal (44% CP)	16.00	10.67	10.67	10.67	10.67	15.00	10.00	10.00	10.00	10.00
Vegetable oil	1.00	1.30	1.30	1.00	1.30	1.00	1.50	1.50	1.00	1.30
Di calcium phosphate	1.80	1.80	1.80	1.80	1.80	1.80	1.80	1.80	1.80	1.80
Ground limestone	2.00	2.00	2.00	2.00	2.00	2.00	2.00	2.00	2.00	2.00
Corn gluten meal	16.50	19.30	18.30	18.50	18.70	12.00	14.50	14.00	14.00	14.00
Common salt	0.30	0.30	0.30	0.30	0.30	0.30	0.30	0.30	0.30	0.30
Vit. and min. premix	0.30	0.30	0.30	0.30	0.30	0.30	0.30	0.30	0.30	0.30
Lysine HCl	0.60	0.60	0.60	0.60	0.60	0.40	0.40	0.40	0.40	0.40
Dl-methionine	00.00	0.00	0.00	00.00	00.00	0.00	00.00	00.00	0.00	0.00
CM	00.00	5.33	0.00	00.00	1.78	0.00	5.00	00.00	0.00	1.67
RSM	00.00	0.00	5.33	00.00	1.78	0.00	00.00	5.00	0.00	1.67
BCM	00.00	0.00	0.00	5.33	1.78	0.00	00.00	00.00	5.00	1.67
Total	100	100	100	100	100	100	100	100	100	100
Energy and nutrients	Calculated analysis (NRC 1994)									
ME (kcal/kg)	3149	3143	3150	3152	3154	3143	3131	3158	3147	3149
Crude protein, CP (%)	23.06	23.03	23.05	23.07	23.04	20.13	20.01	20.26	20.20	20.07
Ether extract, EE (%)	3.88	4.82	4.49	5.09	4.99	3.97	5.07	5.04	5.11	5.04
Crude fiber, CF (%)	2.69	2.72	2.60	2.63	2.64	2.68	2.71	2.57	2.62	2.64
Calcium, %	1.20	1.18	1.18	1.18	1.18	1.20	1.18	1.18	1.18	1.18
Total phosphorus, %	0.70	0.69	0.70	0.69	0.69	0.68	0.68	0.69	0.68	0.68
Available phosphorus, %	0.45	0.54	0.45	0.45	0.45	0.45	0.44	0.44	0.44	0.44
Lysine, %	1.22	1.13	1.17	1.14	1.15	1.10	0.92	0.96	0.93	0.94
Methionine, %	0.46	0.47	0.48	0.47	0.47	0.39	0.41	0.41	0.41	0.41
Methionine + Cystine, %	0.85	0.87	0.88	0.87	0.88	0.74	0.76	0.78	0.76	0.76

Each 3 kg of premix contained vit. A, 12,000 IU; vit. D_3_, 2200 IU; vit. E, 10 mg; vit. K_3_, 2.0 g; vit. B_1_, 1.0 g; vit. B_2_, 5.0 g; vit. B_6_, 1.5 g; vit. B_12_, 10 mg; pantothenic acid, 10 mg; niacin, 30 mg; folic acid, 1.0 g; biotin, 50 mg; choline chloride, 300 mg; Mn, 60 mg; Zn, 50 mg; Cu, 10 mg; Fe, 30 mg; I, 1.0 g; Se, 100 mg; Co, 100 mg; and CaCO_3_, 3g. *CM* coconut meal, *RSM* rocket seed meal, *BCM* black cumin meal, *ME* metabolizable energy

### Preparation of chitosan nanoparticles

For the preparation of nanoparticles of hydrophobic polymers, ionic gelation methods were used. The method of preparation was extremely gentle and involved a mixture of two aqueous phases at room temperature. The first phase, 0.2 g chitosan (Sigma-Aldrich, Egypt), was dissolved with 200 ml distilled water containing 1 ml acetic acid at 25 °C for 30 min. The pH of the mixture was adapted to be 7.2, while in the second phase, 0.066 g polyanion sodium tripolyphosphate was dropped slowly with stirring. Using a Zetasizer, it was shown that the size of nano-chitosan was between 30 and 40 nm. Chitosan nanoparticles coating applications were a sub-major factor of 10 ml/kg diet.

### Performance criteria

Chicks of each replication were weighed at the start of study and then once a week until the end of the study (6 weeks of age). Also, feed intake (FI) and body weight gain (BWG) were estimated weekly on the basis of replicate groups; thus, feed conversion ratio (FCR) was calculated.

### Blood biochemical parameters

During slaughter (6 weeks of age), ten blood samples were collected in heparinized test tubes from chicks of each treatment. Centrifugation was used to separate blood plasma at 3000 rpm for 10 min and stored at – 20 °C until analysis. Plasma concentrations of immunoglobulins (IgG, IgM, and IgA), malondialdehyde (MDA), total antioxidant capacity (TAC), thyroid hormones thyroxin (T4) and triiodothyronine (T3), follicle-stimulating hormone (FSH), and blood plasma activity of catalase (CAT) and superoxide dismutase (SOD) were determined by commercial kits.

### Histological examination of lymphoid organs

Tissue samples from the thymus, bursa of Fabricius, and spleen were carefully dissected and immediately fixed in a 10% formalin solution. As described by Janqueira et al. (1981), the paraffin method was used to prepare permanent sections. Samples were dehydrated in ethyl alcohol solutions with increasing concentrations ranging from 70% to absolute ethanol alcohol. Tissue blocks were created by clearing samples in xylene and embedding them in melted paraffin wax. They were sectioned (4–5-microns thick) and mounted on glass slides before being stained with hematoxylin and eosin. The histological sections were performed at Cairo University’s Pathology Laboratory, Faculty of Veterinary Medicine. Sections were photographed with a digital camera and examined under a light microscope.

## Results and discussion

### Growth performance of broiler chickens

#### Effect of plant protein source

The effects of feeding the diets containing three plant protein sources (coconut meal: CM, rocket seed meal: RSM, and black cumin meal: BCM) and their combination as partial substitutes for soybean meal (SBM) with or without NCH on growth performance of broiler chickens are presented in Table 2. The initial body weight (IBW) of chicks ranged from 44.4 to 44.5 g, with no significant differences (*P* > 0.05) among various experimental groups. At the end of the study (6 weeks of age), chicks fed the diets containing the tested plant protein sources (CM, RSM, BCM, and their combination) displayed significantly (*P* ≤ 0.05) heavier final body weights (FBWs) than that of the control group, regardless of added dietary NCH. Means of FBW for birds fed the diets containing CM, RSM, BCM, and their combination were 2083.2, 2092.5, 2106.9, and 2073.0 g, respectively, compared with1983.2 g for the control chicks. Similarly, birds fed the diets containing CM, RSM, BCM, and their combination achieved significantly better (*P* ≤ 0.05) body weight gain (BWG) for the duration of the experiment (0 to 6 weeks of age) than did the control ones (Table 2). When CM alone or in combination with RSM plus BCM replaced one-third (wt/wt) of dietary SBM the cumulative FI of chicks for the entire experimental period significantly reduced (*P* ≤ 0.01) in comparison to the control group (Table 2). However, the cumulative FI of chicks fed the diets containing RSM or BCM as partial substitutes for SBM (33.3% wt/wt) was measured statistically to be slightly lower (*P* > 0.05) than the control birds but comparable to those fed the diet containing CM + RSM + BCM (Table 2). Independent from added dietary NCH, the cumulative feed conversion ratio (FCR) of broilers fed diets with three plant protein sources (CM, RSM, and BC) and their combination as partial substitutes for SBM was significantly superior (*P* ≤ 0.01) to that of the control chicks.

**Table 2:** Growth performance and blood plasma criteria of immune response for 6-week-old broiler chickens fed diets containing three plant protein sources and their combination as partial substitutes for SBM with or without nano-chitosan (NCH)

Main effects: protein source (A):	IBW (g)	FBW (g)	BWG (g)	FI (g)	FCR (g:g)	IgG (mg/dL)	IgM (mg/dL)	IgA (mg/dL)	TAC (nmol/mL)	MDA (nmol/mL)
Soybean meal (SBM: A1)	44.4	1983.2^b^	1938.7^b^	3222.1^a^	1.67^a^	420.54^d^	174.22^b^	120.28^b^	1.65^b^	29.79^a^
Coconut meal (CM: A2)	44.5	2083.2^a^	2038.7^a^	3031.9^c^	1.49^c^	465.79^c^	185.11^b^	129.07^ab^	1.69^b^	25.36^b^
Rocket seed meal (RSM: A3)	44.5	2092.5^a^	2048.0^a^	3179.9^ab^	1.56^b^	516.56^b^	222.86^a^	138.97^a^	1.88^a^	18.78^d^
Black cumin meal (BCM: A4)	44.4	2106.9^a^	2062.5^a^	3139.2^ab^	1.52^bc^	535.83^b^	229.06^a^	133.83^a^	1.73^b^	21.91^c^
CM+ RSM+ BCM (A5)	44.5	2073.0^a^	2028.5^a^	3126.9^b^	1.54^bc^	565.71^a^	232.37^a^	134.44^a^	1.70^b^	19.27^d^
Standard error of the means	0.016	2.39	2.39	2.98	0.02	9.05	4.75	3.35	0.03	0.78
Significance level	NS	*	*	**	**	*	*	**	*	*
Added NCH (B)										
0.0 g/kg diet (B1)	44.5	2046.6	2002.1	3113.7	1.56	496.91	196.89^b^	130.55	1.69^b^	24.65^a^
1.0 g/kg diet (B2)	44.5	2088.9	2044.4	3166.2	1.55	504.94	220.55^a^	132.09	1.77^a^	21.40^b^
Standard error of the means	0.01	1.51	1.5	1.89	0.01	5.72	3.00	2.12	0.01	0.49
Significance level	NS	NS	NS	NS	NS	NS	*	NS	*	*
A by B interaction										
A1 B1	44.5	1871.2	1826.7	3154.5	1.73	426.90	172.38	144.88	1.66	30.62
A1 B2	44.4	2095.2	2050.8	3289.8	1.61	414.18	176.06	125.68	1.64	28.67
A2 B1	44.5	2036.2	1991.7	3009.0	1.51	472.98	179.78	130.80	1.72	28.59
A2 B2	44.6	2130.3	2085.7	3054.8	1.47	458.60	190.44	127.34	1.65	22.13
A3 B1	44.5	2158.3	2113.8	3209.0	1.52	492.58	204.82	140.82	1.82	20.40
A3 B2	44.6	2026.8	1982.2	3150.8	1.59	540.54	240.90	137.12	1.93	17.18
A4 B1	44.5	2129.8	2085.3	3132.0	1.50	530.84	206.00	134.96	1.65	22.62
A4 B2	44.4	2084.1	2039.7	3146.6	1.55	540.82	252.12	132.70	1.82	21.20
A5 B1	44.5	2037.6	1993.1	3064.4	1.54	561.24	221.48	131.28	1.59	21.01
A5 B2	44.5	2108.5	2064.0	3189.4	1.54	570.58	243.26	137.60	1.82	17.54
Standard error of the means	0.02	3.38	3.38	4.22	0.02	12.81	6.72	4.74	1.66	1.10
Significance level	NS	NS	NS	NS	NS	NS	NS	NS	NS	NS

Each criterion means within the same column having different superscripts differ significantly (*P* ≤ 0.05). *NS*: not significant. *: significant at *P* ≤ 0.05. **: significant at *P* ≤ 0.01. *IBW*: initial body weight. *FBW*: final body weight. *FI*: feed intake. *FCR*: feed conversion ratio. IgG, IgM, and IgA are immunoglobulins G, M, and A, respectively. *TAC* total antioxidant capacity, *MDA* malondialdehyde

Although the experimental diets, applied herein, were isocaloric (Table 1), the observed reduction in cumulative FI of broiler chickens fed the diets containing CM alone or in combination with RSM plus BCM as partial substitutes for one-third of SBM in the control diet might be attributed, at least partly, to a higher EE contents in the diets containing the tested plant protein sources compared with the control diet. The EE contents of starter and finisher diets containing the tested plant proteins (CM, RSM, BCM, and their combination) ranged from 4.49 to 5.09% and from 5.04 to 5.11% compared with 3.88% and 3.97% for the control corn-soybean meal, respectively (Table 1). It is generally accepted that feeding high-fat diets is associated with a reduced rate at which feed passes through the gastrointestinal tract of birds, leading to impairing their FI. The noticed improvements in FBW, BWG, and FCR of broilers fed the tested plant proteins (coconut, recuta seed, and black seed meals) applied herein are consistent with the results obtained by Abou El-Maaty et al. (2021). This is attributed to the high content of different essential amino acids that existed in tested meals compared to control meal as illustrated in Tables S1, 2 and 3. They found that broiler chickens fed the diets containing RSM (7.5%) during the starter and finisher phases of growth achieved superior means of FBW, BWG, and FCR to those of the control group. Similarly, Abdo (2003) found that broiler chickens fed diets in which RSM replaced 10 and 25% of dietary SBM exhibited the best means of BWG compared with their control group. Growth performance (FBW, BWG and FCR) of broilers was improved due to feeding diets enriched with rocket seed oil (1.0 g/kg) in comparison to the control group (Razooqi et al. 2014).

Working with growing rabbits, El-Tohamy and El-Kady (2007) found a significant increment of BWG and FI, and an improvement in FCR when RSM replaced 50% of dietary SBM. On the contrary, feeding the growing pullets on a diet containing CM (200 g/kg) depressed their efficiency of feed utilization (Diarra et al. 2018). Also, Osman et al. (2004) reported that feeding RSM as a partial substitute (15%) for dietary SBM had no effect on broiler chicken live body weight. In addition, Mael et al. (2019) observed no adverse effect on the performance of growing pullet when fed diets containing two levels of CM (200 or 300 g/kg) from 6 to 18 weeks of age. On the other hand, the enhanced growth performance of broilers in the present study is in agreement with the findings of Abou El-Soud (2000) who found that feeding diet containing 2.0% BCM caused an improvement in BWG, FI, and FCR of growing quails compared with the control group. With growing rabbits, El-Nomeary et al. (2015) found that feeding diets containing 3.0% BCM for 68 days improved FBW, BWG, and FCR compared with the control group. However, Khan, and S.H., J. Ansari, A. Haq and G. Abbas. (2012) concluded that the inclusion of BCM up to 5.0% in diets of broiler chickens has no negative consequences for their growth performance.

#### Effect of added NCH

Apart from a plant protein source, broiler chickens fed the diets enriched with nano-chitosan exhibited comparable means of FBW, BWG, and FCR to those fed the control diets, with no significant differences among them (Table 2). This result is consistent with the findings of Hassan et al. (2021), who found no positive effect of feeding NCH-fortified (30 or 50 mg/kg) diets on growth performance of Japanese quail. With laying hens, Hamady and Farroh (2020) reported that feeding diets supplemented with NCH (50 or 200 mg/kg) did not significantly affect rate of egg production, egg weight, and FCR from 23 to 34 weeks of age. In disagreement with our results, Abou El-Maaty et al. (2021) demonstrated that broilers fed NCH-supplemented diets achieved better FCR, FBW, and BWG along with the least mean of FI than did their control counterparts. Also, El-Ashram et al. (2020) observed a beneficial feeding effect NCH-enriched effects of diet on growth performance (FBW, BWG, and FCR) of Japanese quail but FI and mortality rates were not affected.

#### Plant protein source by added NCH interaction

Data summarized in Table 2 indicated that the growth performance (FBW, BWG, and FCR) of broiler chickens was not significantly affected (*P* > 0.05) because of the interaction between dietary plant protein source and nano-chitosan addition.

### The immune response of broiler chickens

#### Effect of plant protein source

The blood plasma criteria of immune response for 6-week-old broiler chickens as affected by feeding diets containing three plant protein sources (CM, RSM, and BCM) and their combination as partial substitutes for SBM with or without NCH are shown in Table 2. Independent from added NCH, birds fed the diets containing the tested plant protein sources (CM, RSM, BCM, and their combination) showed significantly greater (*P* ≤ 0.05) plasma concentrations of IgG compared to the control group. Significantly greater plasma levels of IgM (*P* ≤ 0.05) and IgA (*P* ≤ 0.01) were recorded for birds fed the diets containing RSM, BCM, and the mixture of tested proteins (CM, RSM, and BCM) compared with their control counterparts or those fed the diet containing CM, with no significant differences between them. Interestingly, chicks fed the diet containing RSM exhibited significantly higher (*P* ≤ 0.05) plasma concentrations of TAC compared with those of the control. Furthermore, the TAC analysis exhibited no significant differences in other groups regardless plant protein source. However, broiler chickens fed the diets containing the tested plant protein sources (CM, RSM, BCM, and their combination) displayed significantly less (*P* ≤ 0.05) plasma concentrations of MDA than did the control group. The observed increase in plasma concentrations of immunoglobulins (IgG, IgM, and IgA) and TAC in broilers fed the tested plant proteins is in line with those of El-Kashef (2020). He found that chicks fed diets containing 3 or 6% black cumin seed showed increased levels of blood globulin and leukocyte count in comparison to the control group. Similar outcomes were obtained by Abou El-Maaty et al. (2021), who stated that broilers fed RSM-containing diets displayed significantly higher serum levels of immunoglobulins (IgG, IgM, IgA) and TAC in comparison to the control group. In addition, El-Bahr et al. (2021) demonstrated that the antibody titers for Newcastle disease (ND) virus and infectious bronchitis (IB) virus were significantly higher in broiler chickens fed a diet supplemented with black cumin seed than in control chickens during 15–28 and 29–42 days of age. Other previous studies have shown that *Nigella sativa* seeds have immuno-stimulating properties that enhance the health status of broilers (Tollba and Hassan 2003; Al-Beitawi and El-Ghousein 2008). Also, Attia and Al-Harthi (2015) documented a significant increase in antibody titer to ND virus and IB disease due to feeding diet containing *Nigella sativa* seed (1.5 g/kg) compared to the negative control group.

#### Effect of added NCH

The impact of dietary supplements with NCH on the immune response of broiler chickens fed the tested plant protein sources (CM, RSM, BCM, and their combination) is given in Table 2. Aside from dietary plant protein source, feeding the NCH-fortified diets to broiler chickens led to significant increases (*P* ≤ 0.05) in plasma levels of IgM and TAC, but the level of MDA decreased significantly (*P* ≤ 0.05) compared with those of the control ones, whereas plasma concentrations of IgG and IgA were not affected. According to the current findings, Xu et al. (2020) reported that enriching diets of weaning pigs with NCH (200 or 400 mg/kg) resulted in an increase in plasma concentration of IgG at 28 days of age, but the level of IgM was not affected. In line with our outcomes, Hassan et al. (2021) reported that dietary fortification with NCH (30 or 50 mg/kg) caused a significant rise in plasma concentrations of TAC of Japanese quails compared with the control birds. Consistent also with our results, Hamady and Farroh (2020) demonstrated that laying hens fed NCH-enriched diets (200 mg/kg) showed a significant reduction in egg yolk contents of MDA in comparison to the control hens. However, Abou El-Maaty et al. (2021) discovered that broiler chickens fed NCH-supplemented diets (1.0 g/kg) exhibited comparable plasma concentrations of immunoglobulins (IgG, IgM and IgA), TAC, and MDA to those of the control chicks.

#### Plant protein source by added NCH interaction

As presented in Table 2, the interaction between dietary plant protein source and NCH addition had no discernible impact (*P* > 0.05) on the concentrations of plasma of immunoglobulins (IgG, IgM, and IgA), MDA, or TAC of broiler chickens. This may be interpreted as the effects of tested plant proteins and NCH were not interrelated.

### Blood plasma antioxidant enzymes and selected hormones

#### Effect of plant protein source

The blood plasma activity of antioxidant enzymes (CAT and SOD), and thyroid hormone levels (T3 and T4) and FSH of 6-week-old broiler chickens as affected by feeding diets containing three plant protein sources (CM, RSM, and BCM) and their combination as partial substitutes for SBM with or without NCH are shown in Table 3. Aside from NCH addition, broilers fed the diets containing the tested plant protein sources (CM, RSM, BCM, and their combination) demonstrated significantly higher (*P* ≤ 0.05) plasma activity of the antioxidant enzymes (CAT and SOD) and levels of FSH than their control counterparts. Plasma T4 levels were significantly higher (*P* ≤ 0.01) in birds fed the diets containing RSM, BCM, and the mixture of the tested proteins compared with their control counterparts or those fed the diet containing CM, with no significant differences between them. Feeding the diets containing the tested plant protein sources and their combination led to a significant increase (*P* ≤ 0.01) in plasma T3 concentrations compared with that of the control group. Our results agree with those of Abou El-Maaty et al. (2021), who reported significant increases in serum concentrations of the antioxidant enzymes (CAT and SOD) and FSH and T3 hormones in broiler chickens fed diets containing 7.5% RSM compared with their control birds. Consistent with the present results, Hassan (2021) found that feeding diets containing *Nigella sativa* seeds to broiler chicks caused a significant increase in blood plasma activity of catalase and glutathione reductase compared with the control group. It is well-known that thyroid hormones are vital regulators of metabolic processes and that T3 is the active form of these hormones. It has been validated that aerobic metabolic processes produce reactive oxygen species (ROS) and there are two endogenous systems (antioxidant enzymes including SOD, CAT, and glutathione peroxidase and non-enzymatic substances including lipid-soluble and water-soluble antioxidants) cooperate to regulate their production (Patekar et al. 2013; Surai et al. 2019). Oxidative stress occurs when the generation of ROS exceeds the antioxidant system’s scavenging capacity (Surai and Fisinin 2015; Sack et al. 2017). Thus, the observed increase in serum level of T3 and activity of CAT and SOD, reported herein, for broilers fed the diets containing the tested plant protein sources could be an indication to high metabolic rate and high potential of the antioxidant enzyme system, resulting in normal oxidative status.

**Table 3:** Blood plasma activity of antioxidant enzymes and levels of thyroid hormones and follicle-stimulating hormone of broiler chickens fed diets containing three plant protein sources and their combination as partial substitutes for SBM with or without nano-chitosan

Main effects: protein source (A):	CAT (U/ml/h)	SOD(U/ml/h)	T4 (IU/mL)	T3(IU/mL)	FSH (IU/mL)
Soybean meal (SBM: A1)	53.14^c^	61.28^c^	19.72^c^	3.40^c^	1.98^c^
Coconut meal (CM: A2)	59.09^b^	76.45^b^	21.39^bc^	4.52^b^	2.76^b^
Rocket seed meal (RSM: A3)	71.21^a^	83.60^a^	22.80^ab^	4.91^ab^	4.52^a^
Black cumin meal (BCM: A4)	68.05^a^	82.22^a^	21.57^ab^	4.48^b^	4.38^a^
CM+ RSM+ BCM (A5)	68.61^a^	86.11^a^	23.27^a^	5.11^a^	4.61^a^
Standard error of the means	1.37	1.48	0.60	0.16	0.10
Significance level	*	*	**	**	*
Added nano-chitosan (B)					
0.0 g/kg diet (B1)	62.11^b^	76.01^b^	21.98	4.36	3.48^b^
1.0 g/kg diet (B2)	65.92^a^	79.85^a^	21.52	4.61	3.82^a^
Standard error of the means	0.86	0.94	0.38	0.10	0.06
Significance level	*	*	NS	NS	*
A by B interaction:					
A1 B1	47.16	58.68	18.27	3.02	2.14
A1 B2	59.12	63.88	21.18	3.78	1.82
A2 B1	56.76	78.40	22.44	4.60	2.53
A2 B2	61.42	74.50	20.33	4.45	2.98
A3 B1	68.40	79.54	23.74	4.70	3.94
A3 B2	74.02	87.66	21.87	5.12	5.11
A4 B1	68.12	81.26	22.20	4.44	4.45
A4 B2	67.98	83.18	20.93	4.53	4.31
A5 B1	70.14	82.18	23.25	5.04	4.32
A5 B2	67.08	90.04	23.30	5.17	4.91
Standard error of the means	1.93	2.10	0.85	0.23	0.15
Significance level	NS	NS	NS	NS	NS

Each criterion means within the same column having different superscripts differ significantly (*P* ≤ 0.05). *NS*: not significant. *: significant at *P* ≤ 0.05. **: significant at *P* ≤ 0.01. *CAT* catalase, *SOD* superoxide dismutase, *T4* thyroxin, *T3* triiodothyronine, *FSH* follicle-stimulating hormone

#### Effect of added NCH

With the exception of plant protein source, broilers fed the diets enriched with NCH displayed significantly greater (*P* ≤ 0.05) plasma activities of CAT and SOD and FSH concentrations than their control counterparts, whereas plasma concentrations of thyroid hormones (T3 and T4) were not affected. Our findings are partially consistent with the findings of Abou El-Maaty et al. (2021), who demonstrated that feeding NCH-enriched diets to broiler chickens resulted in significant increases in serum concentrations of T3 and FSH compared with the control birds but T4 level and activity of SOD were not affected.

#### Plant protein source by added NCH interaction

The interaction between dietary plant protein source and NCH addition had no discernible impact (*P* > 0.05) on plasma levels of thyroid hormones (T3 and T4), FSH, or activity of antioxidant enzymes (CAT and SOD).

### Histological observations on lymphoid organs of broiler chickens

#### Histological structure of thymus

**Fig. 1:**
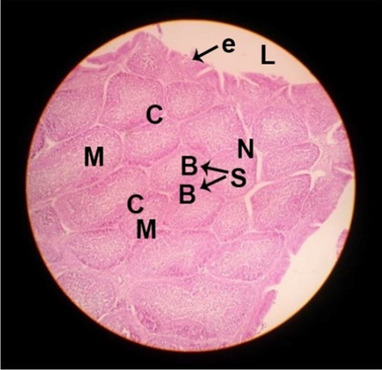
Histological structure of the thymus from the control group

**Fig. 2:**
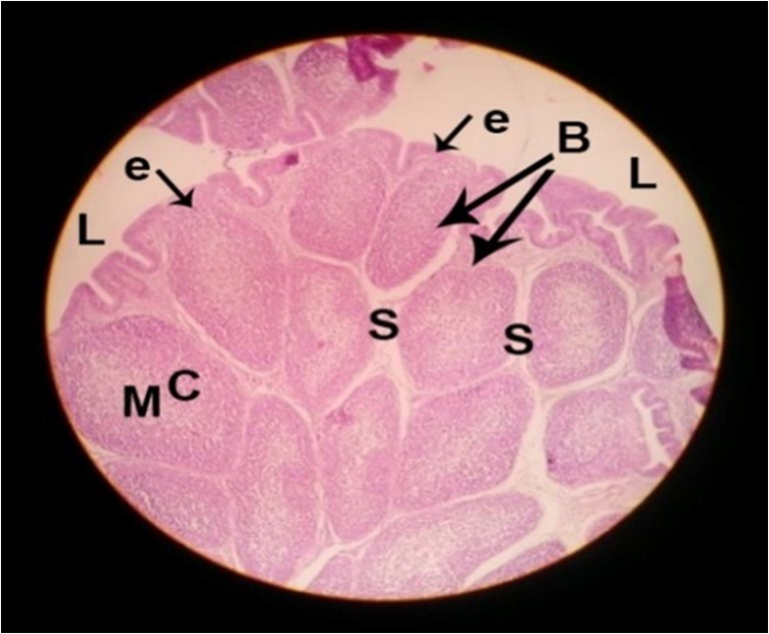
Histological structure of the thymus from chicks fed CM

**Fig. 3:**
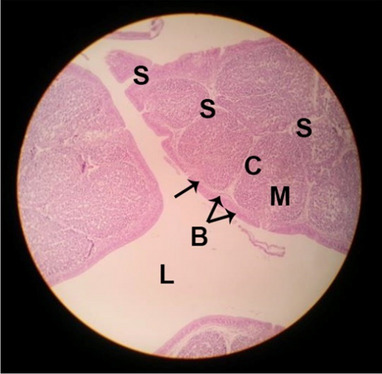
Histological structure of the thymus from chicks fed RSM

**Fig. 4:**
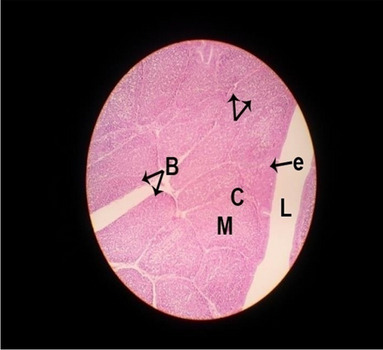
Histological structure of the thymus from chicks fed BCM

**Fig. 5:**
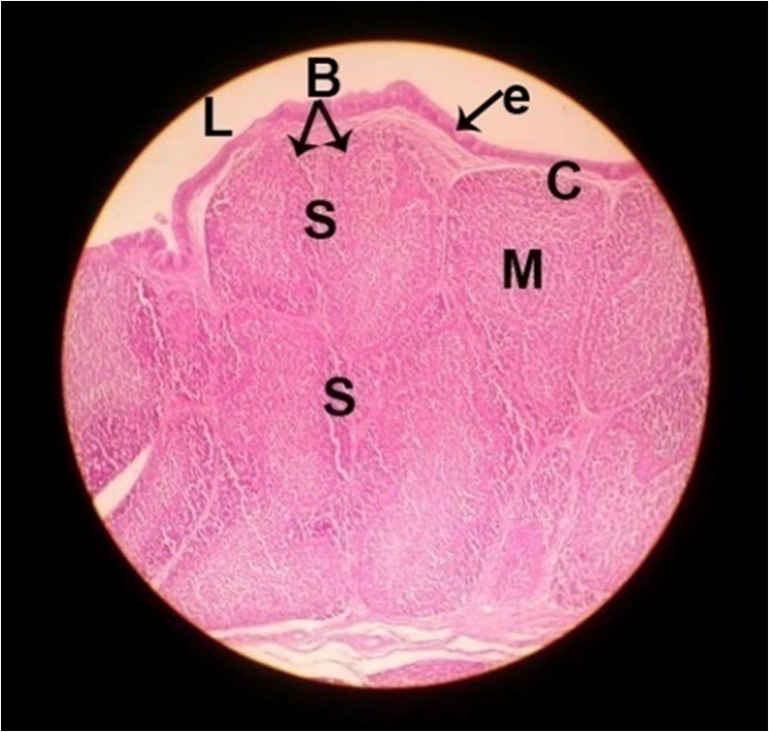
Histological structure of the thymus from chicks fed the mixture of CM, RSM, and BCM

**Fig. 6:**
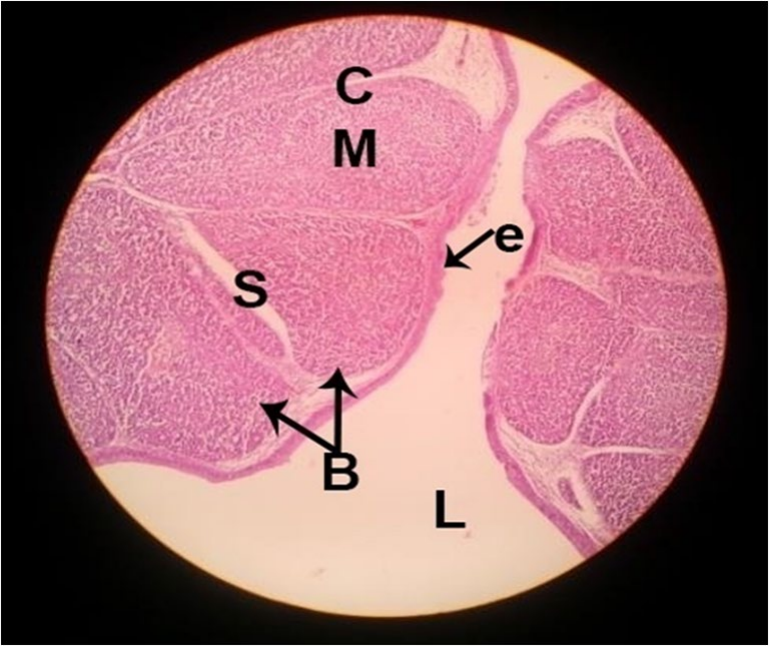
Histological structure of the thymus from chicks fed SBM + nano-chitosan

**Fig. 7:**
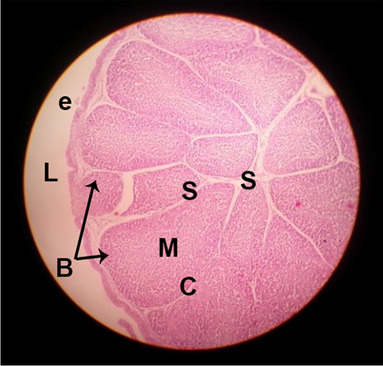
Histological structure of the thymus from chicks fed CM + nano-chitosan

**Fig. 8:**
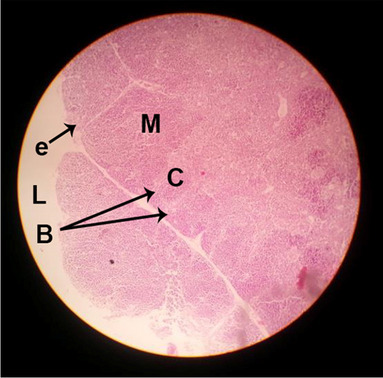
Histological structure of the thymus from chicks fed RSM + nano-chitosan

**Fig. 9:**
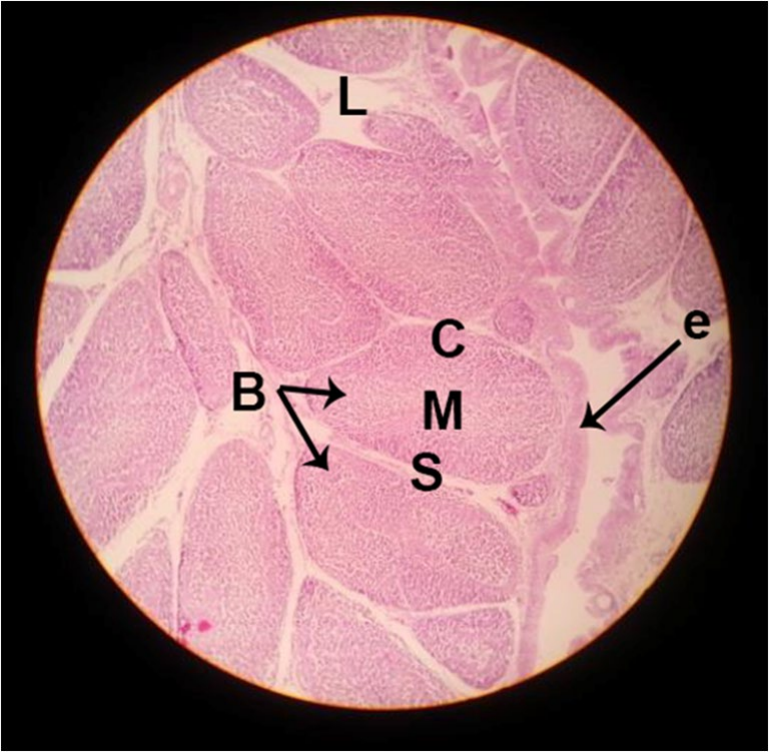
Histological structure of the thymus from chicks fed BCM + nano-chitosan

**Fig. 10:**
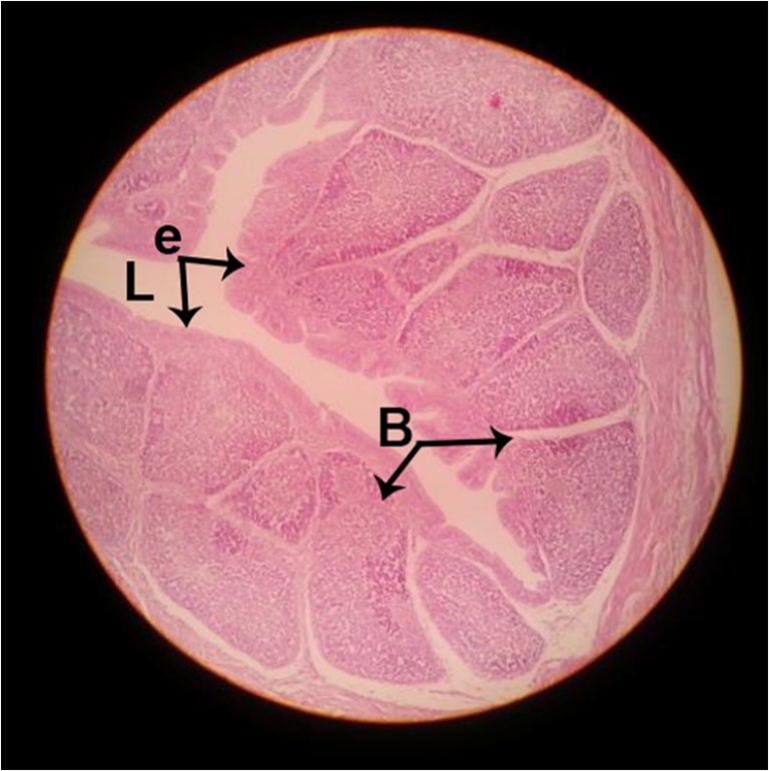
Histological structure of the thymus from chicks fed a mixture of CM, RSM, and BCM + nano-chitosan

The consequences of feeding diets containing three plant protein sources (coconut meal: CM, rocket seed meal: RSM, and black cumin meal: BCM) and their combination as partial substitutes for soybean meal (SBM) with or without nano-chitosan on the histological structure of thymus gland of broiler chickens are illustrated in ten plates (Figs. 1, 2, 3, 4, 5, 6, 7, 8, 9, and 10). In general, thymus section from the control chicks (Fig. 1) showed fine connective tissue septa(s) which divide the gland into lobules (T) or segments. Within each lobule, there are large lymphocytes (l), especially in the dark-stained cortex region (c). The medullary region (M) is pale or merged in the cortex area. It is clearly observed that the thymic lobules differ in size, number, and shape due to the treatments. In this concern, the number of lobules were greater in thymus of chicks illustrated in Figs. 2, 4, 8, 9, and 10. However, the lobule size was larger in Figs. 3, 5, 6, 7, 8, and 9. Moreover, the cortex area showed lymphocytes differing in their size from small to large cells, giving rise to dark starred area, especially in sections illustrated in Figs. 4, 5, 6, 7, 8, 9, and 10. This is a good indicator for better immune response of the chick treated with CM, RSM, BCM, and their combination. These observed changes in thymus sections may be resulted from the fact that the immune response of chicks at older ages depends mainly on B-lymphocytes production from the bursa of Fabricius gland, than the T-lymphocytes. This was confirmed by the results obtained by Akter et al. (2006), who observed no changes in lymphoid organs.

#### Histological structure of bursa of Fabricius

**Fig. 11:**
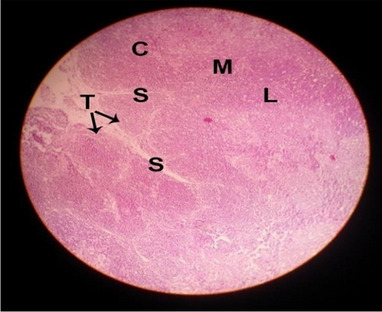
Histological structure of the bursa from the control group

**Fig. 12:**
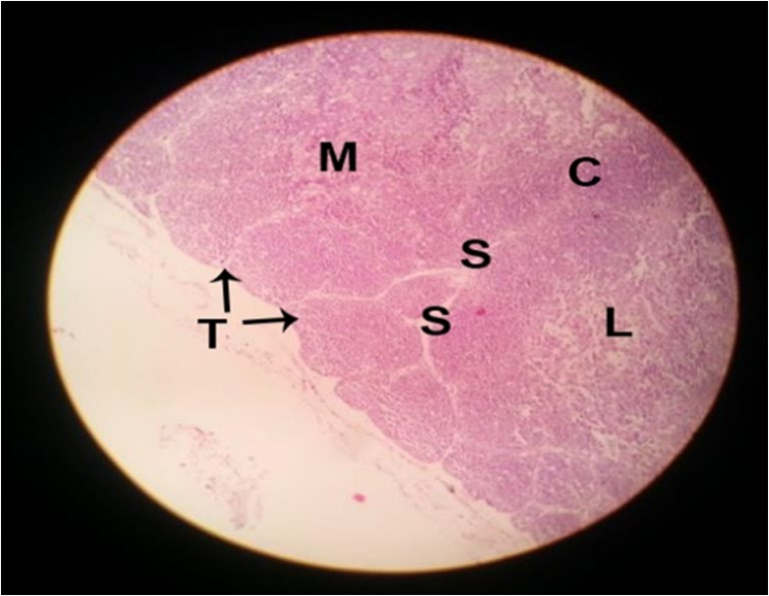
Histological structure of the bursa from chicks fed CM

**Fig. 13:**
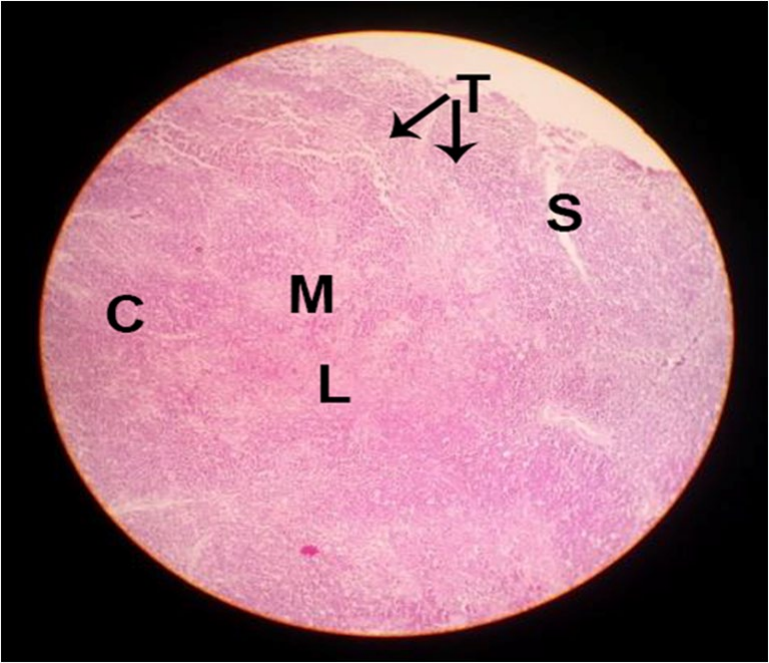
Histological structure of the bursa from chicks fed RSM

**Fig. 14:**
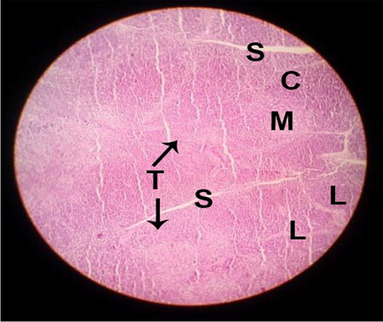
Histological structure of the bursa from chicks fed BCM

**Fig. 15:**
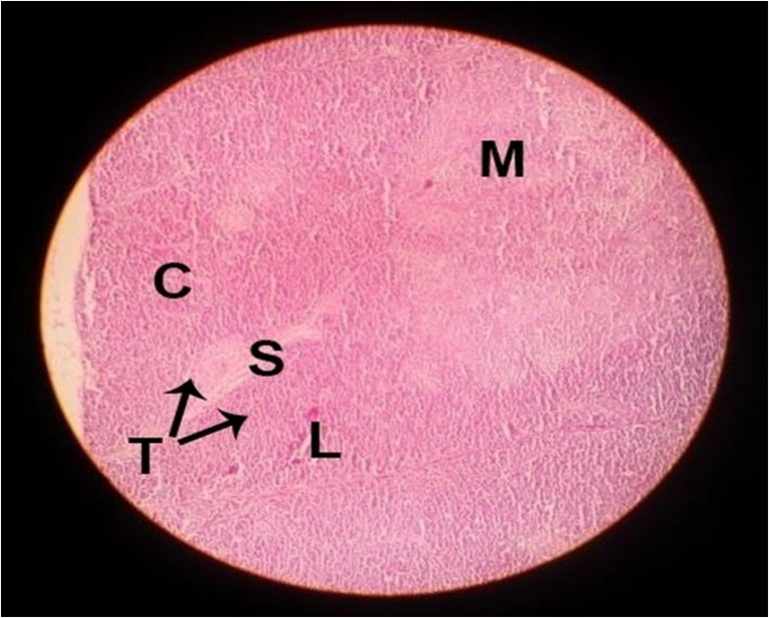
Histological structure of the bursa from chicks fed the mixture of CM, RSM, and BCM

**Fig. 16:**
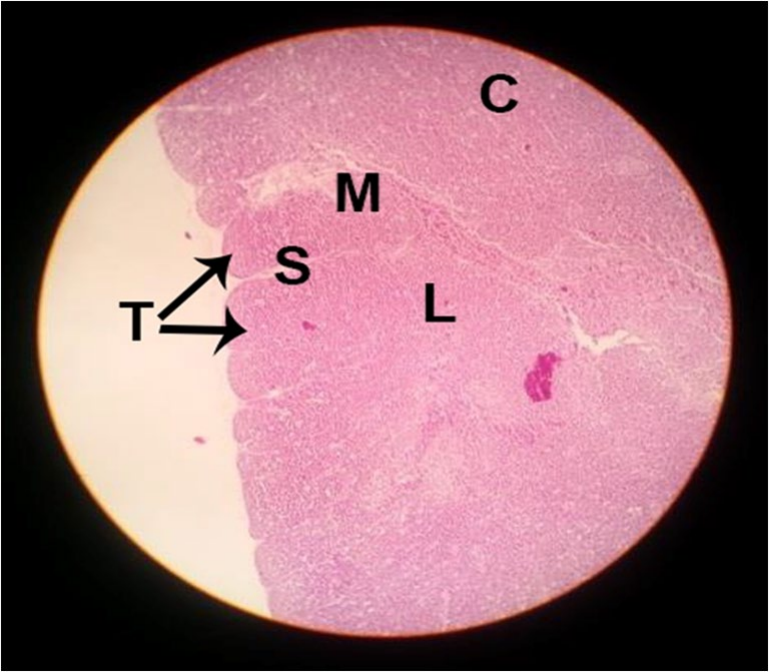
Histological structure of the bursa from chicks fed SBM + nano-chitosan

**Fig. 17:**
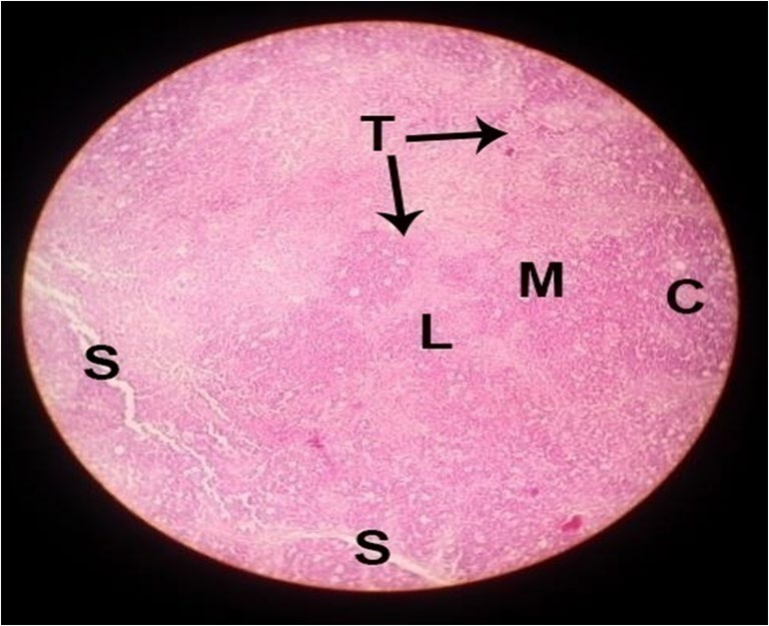
Histological structure of the bursa from chicks fed CM + nano-chitosan

**Fig. 18:**
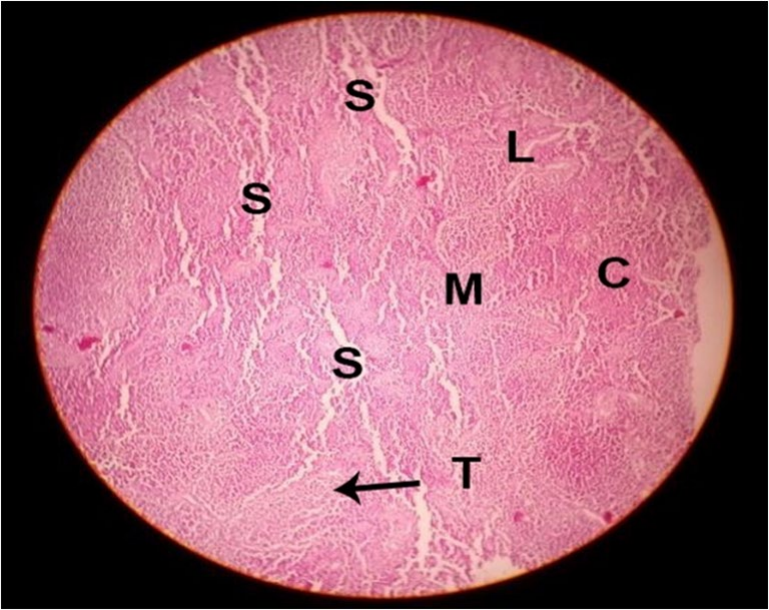
Histological structure of the bursa from chicks fed RSM + nano-chitosan

**Fig. 19:**
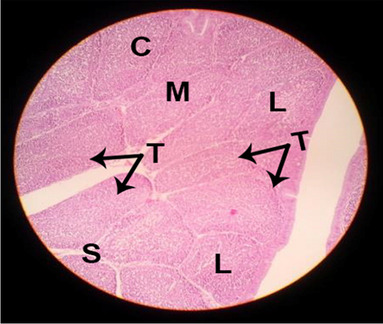
Histological structure of the bursa from chicks fed BCM + nano-chitosan

**Fig. 20:**
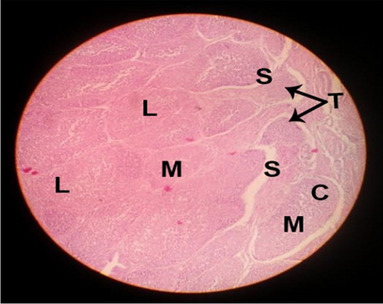
Histological structure of the bursa from chicks fed the mixture of CM, RSM, and BCM + nano-chitosan

The effects of feeding the diets containing three plant protein sources (coconut meal: CM, rocket seed meal: RSM, and black cumin meal: BCM) and their combination as partial substitutes for soybean meal (SBM) with or without nano-chitosan on the histological structure of bursa of Fabricius of broiler chickens are illustrated in ten plates (Figs. 11, 12, 13, 14, 15, 16, 17, 18, 19, and 20). The histological analysis of bursa sections of different treatments indicates that the basic histological structure of the bursa tissues is nearly similar (Figs. 11, 12, 13, 14, 15, 16, 17, 18, 19, and 20). The bursa is well known to be a primary lymphoid organ in birds, and it appears as 20-fold; each of them contains numerous follicles (B) varied in their size. These follicles are divided into two sections, cortex (c) and medulla (M), enclosed in a pseudo-stratified columnar epithalamic outer layer as shown in all sections, especially in Figs. 11, 12, 13, 15, 16, 17, 19, and 20. There are many fine septates (s) that separate these follicles from each other, while their secretions are collected in lumens (l). It was clearly observed that the cortex is more clear because it is made up of many small lymphocytes; it was more stained than the medulla. This structure was clearly shown in all bursa sections either the control or other treatments. However, the number of bursa follicles was greater in Fig. 11 (control) followed by Figs. 12, 17, and 19. On the other hand, the effect of dietary treatments revealed a positive influence on the histological structure of bursa in terms of increased size of bursal follicles and very well-arranged epithelial lining (Figs. 15, 16, 17, 19, and 20) along with a narrow connective tissue septa between follicles compared by the control chicks. Also, it was observed that the improved bursal histology is associated by an obvious increase in the cortex area with apparently ketter epithelium covering of the whole plicae (Figs. 13, 15, 16, 17, 18, and 20). It is likely that under the prevalent conditions of the present study, the applied treatments exerted beneficial effects on bursal function via their effect on promoting immunoglobulins secretion as the number of lymphocytes increased. This was clarified in sections from birds in treatments illustrated in Figs. 12, 14, 15, 16, 17, 18, and 20.

#### Histological structure of spleen

**Fig. 21:**
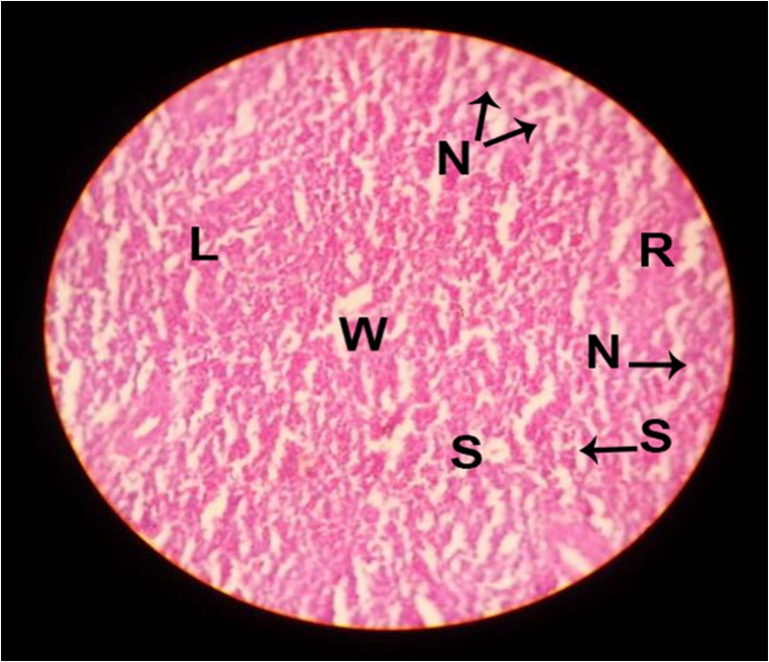
Histological structure of the spleen from the control group

**Fig. 22:**
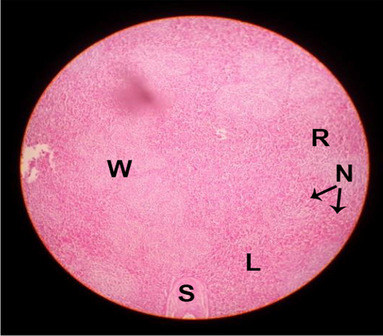
Histological structure of the spleen from chicks fed CM

**Fig. 23:**
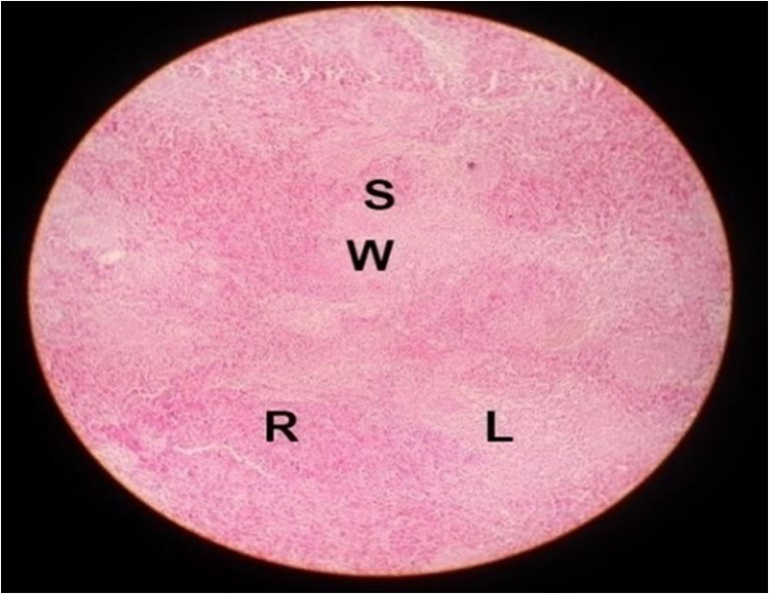
Histological structure of the spleen from chicks fed RSM

**Fig. 24:**
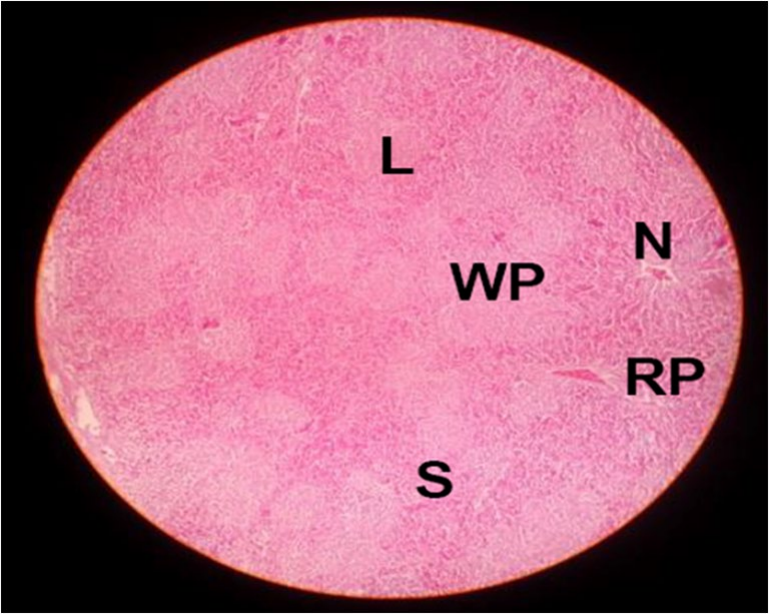
Histological structure of the spleen from chicks fed BCM

**Fig. 25:**
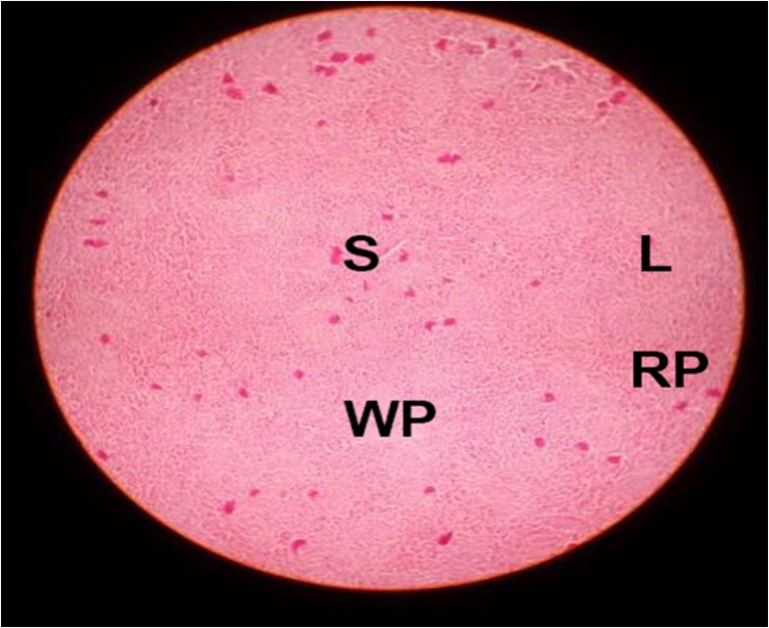
Histological structure of the spleen from chicks fed the mixture of CM, RSM, and BCM

**Fig. 26:**
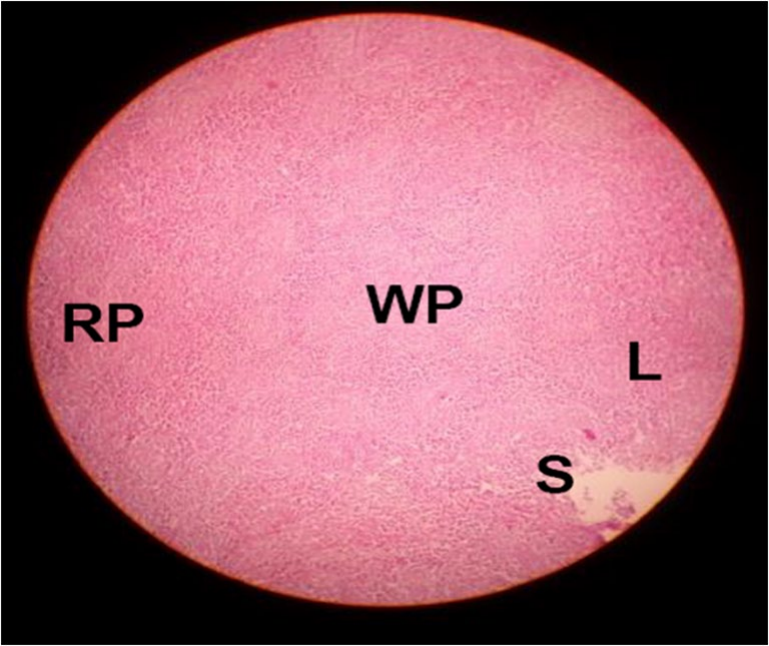
Histological structure of the spleen from chicks fed SBM + nano-chitosan

**Fig. 27:**
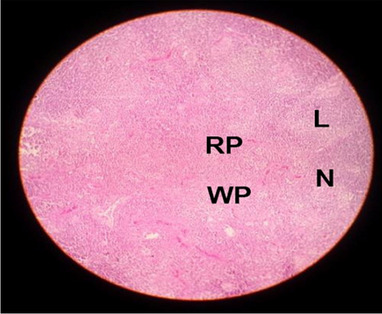
Histological structure of the spleen from chicks fed CM + nano-chitosan

**Fig. 28:**
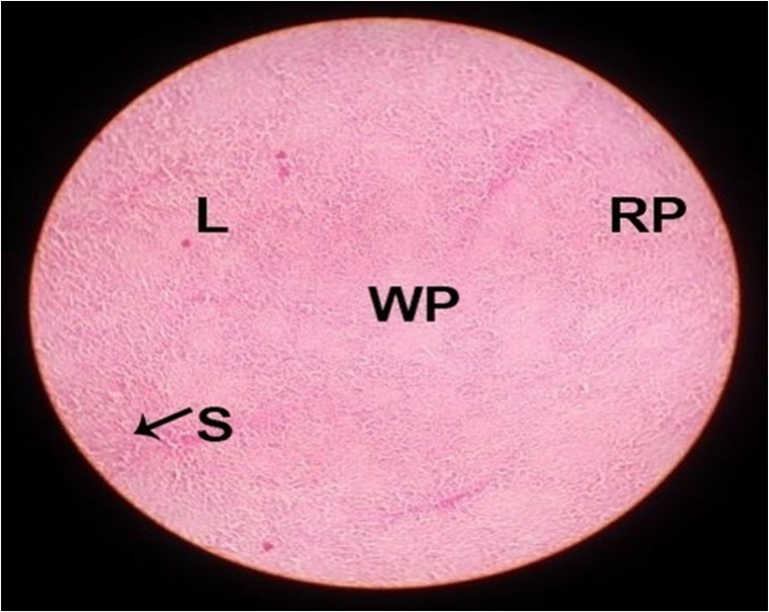
Histological structure of the spleen from chicks fed RSM + nano-chitosan

**Fig. 29:**
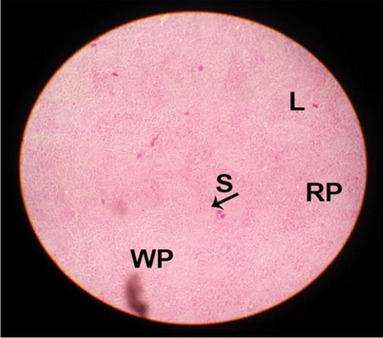
Histological structure of the spleen from chicks fed BCM + nano-chitosan

**Fig. 30:**
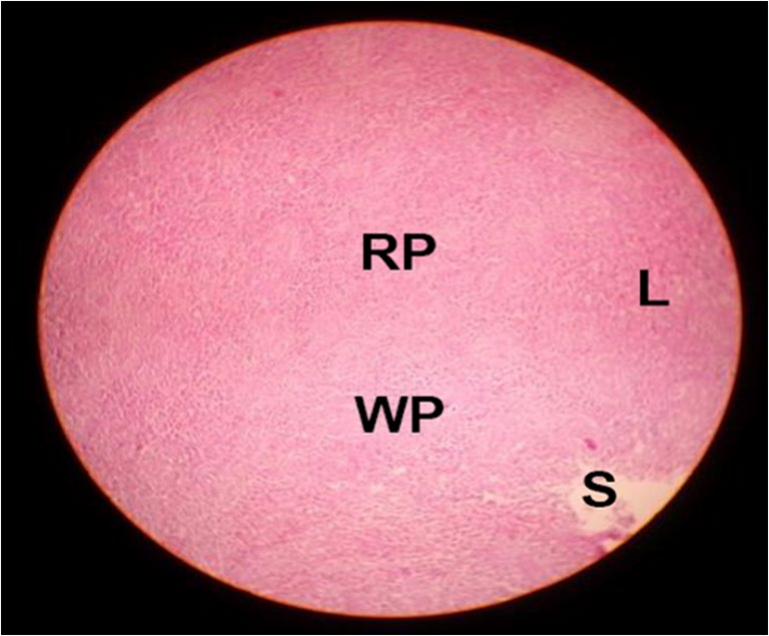
Histological structure of the spleen from chicks fed the mixture of CM, RSM, and BCM + nano-chitosan

The effects of feeding the diets containing three plant protein sources (coconut meal: CM, rocket seed meal: RSM, and black cumin meal: BCM) and their combination as partial substitutes for soybean meal (SBM) with or without nano-chitosan on the histological structure of spleen of broiler chickens are illustrated in ten plates (Figs. 21, 22, 23, 24, 25, 26, 27, 28, 29, and 30). The basic structure of the spleen as shown in these sections is the presence of two main areas, i.e., a large area of white pulp (WP) and a dark-stained red pulp (RP) with numerous blood capillaries, sinusoids (s), and more or less lymphatic nodules (n). This was observed in the spleen sections from the control (Fig. 21) chicks; however, in the other sections, an increase in RP area was noticed in Figs. 22, 24, 26, 27, and 30 compared with other treatments. Moreover, there was an irregular distribution of the RP area within the WP area, especially in the sections from treatments illustrated in Figs. 23, 24, 25, 27, 28, and 30. Since the RP area is expended throughout the splenic sections with a marked increase in the number of large lymphocytes, basophilic hemosiderin granules are found between the blood sinuses (s). These large-sized lymphocytes may reflect an improvement in the immune response of chicks from these treatment groups. Many lymph nodules could be seen in the same sections (Figs. 21, 22, and 23) which may be due to an increase in the differentiation of small lymphocytes to more larger ones. From the previous findings, it appears that there is an evidence to confirm that the spleen of birds harbors large number of T- and B-cells which differentiate into antigen.

## Conclusion

It can be concluded that coconut meal, rocket seed meal, and black cumin meal can be used as dietary plant proteins alone or in combination with nano-chitosan, without affecting broiler chicken growth performance, blood parameters, or immune response.

### Supplementary information

ESM 1 (DOCX 16 kb)

## Data Availability

The data that support the findings of this study are available from the corresponding author, (A. H. Mansour), upon reasonable request.
