# Supplementary material for: Interactive effects of dietary protein and nano-chitosan on growth performance, immune response, and histological aspects of lymphoid organs in broiler chickens
Source: Trop Anim Health Prod. 2024 Jan 27;56(2):62. doi: 10.1007/s11250-023-03855-2 (PMC10817830; doi:10.1007/s11250-023-03855-2)
Supplement: Supplementary file 1 — (DOCX 16 kb) [file 11250_2023_3855_MOESM1_ESM.docx]

**INTERACTIVE EFFECTS OF DIETARY PROTEIN AND NANO-CHITOSAN ON GROWTH PERFORMANCE, IMMUNE RESPONSE, AND HISTOLOGICAL ASPECTS OF LYMPHOID ORGANS IN BROILER CHICKENS**

A.H. Mansour^1*^, M.H. Rabie^1^, Eman A. El-Said^2^ and Hayam A. Abo El-Maaty^1*^

^1^Poultry Production Department, Faculty of Agriculture, Mansoura University, Egypt.

^2^Poultry Production Department, Faculty of Agriculture, Damietta University, Egypt.

Corresponding author’s E-Mail address: awadhany2@outlook.com

**Table (S1) Amino acids of Coconut Meal:**

| **Amino acid** | **%** | **Amino acid** | **%** |
| --- | --- | --- | --- |
| Aspartic (ASP) | 1.18 | Tyrosine (TYR) | 0.43 |
| Threonine (THR) | 0.48 | Phenylalanine (PHE) | 0.66 |
| Serine (SER) | 0.65 | Histidine (HIS) | 0.32 |
| Glutamic (GLU) | 2.63 | Lysine (LYS) | 0.55 |
| Glycine (GLY) | 0.61 | Arginine (ARG) | 2.12 |
| Alanine (ALA) | 0.77 | Proline (PRO) | 0.43 |
| Valine (VAL) | 0.96 | Cystine (CYS) | 0.23 |
| Isoleucine (ILE) | 0.57 | Methionine | 0.29 |
| Leucine (LEU) | 0.94 |  |  |

**Table (S2) Amino acids of Rocker seed Meal:**

| **Amino acid** | **%** | **Amino acid** | **%** |
| --- | --- | --- | --- |
| Aspartic (ASP) | 1.96 | Tyrosine (TYR) | 0.90 |
| Threonine (THR) | 1.03 | Phenylalanine (PHE) | 1.12 |
| Serine (SER) | 1.13 | Histidine (HIS) | 0.75 |
| Glutamic (GLU) | 4.12 | Lysine (LYS) | 1.29 |
| Glycine (GLY) | 1.21 | Arginine (ARG) | 2.03 |
| Alanine (ALA) | 1.37 | Proline (PRO) | 1.74 |
| Valine (VAL) | 1.66 | Cystine (CYS) | 0.64 |
| Isoleucine (ILE) | 1.05 | Methionine | 0.46 |
| Leucine (LEU) | 1.73 |  |  |

**Table (S3) Amino acids of Nigella Sativa seed Meal:**

| **Amino acid** | **%** | **Amino acid** | **%** |
| --- | --- | --- | --- |
| Aspartic (ASP) | 1.40 | Tyrosine (TYR) | 0.71 |
| Threonine (THR) | 0.70 | Phenylalanine (PHE) | 0.69 |
| Serine (SER) | 0.79 | Histidine (HIS) | 0.49 |
| Glutamic (GLU) | 3.00 | Lysine (LYS) | 0.68 |
| Glycine (GLY) | 0.91 | Arginine (ARG) | 1.62 |
| Alanine (ALA) | 0.82 | Proline (PRO) | 0.96 |
| Valine (VAL) | 0.80 | Cystine (CYS) | 0.36 |
| Isoleucine (ILE) | 0.65 | Methionine | 0.4 |
| Leucine (LEU) | 1.03 |  |  |
